# Morphological and Molecular Evidence for a New Species Within *Styrax* (Styracaceae) from a Karst Area in Southwest Guangxi, China

**DOI:** 10.3390/plants14121789

**Published:** 2025-06-11

**Authors:** Guoxing Peng, Tongjun Liang, Jipeng Liang, Yitian Wang, Liaocheng Zhao, Rui Zhang, Yusong Huang, Zhi Li, Weibin Xu, Ming Tang

**Affiliations:** 1 Jiangxi Provincial Key Laboratory of Improved Variety Breeding and Efficient Utilization of Native Tree Species, Jiangxi Agricultural University, Nanchang 330045, China; p_guoxing@163.com (G.P.); wangyitian1999@163.com (Y.W.); 2 Lushan Botanical Garden, Jiangxi Province and Chinese Academy of Sciences, Jiujiang 332900, China; liangtongjun7522@163.com; 3 Management Center of Chongzuo White-Headed Langur National Nature Reserve in Guangxi, Chongzuo 532200, China; ljp995326@126.com; 4 Laboratory of Systematic Evolution and Biogeography of Woody Plants, College of Ecology and Nature Conservation, Beijing Forestry University, Beijing 100083, China; zhaoliaochengzlc952@bjfu.edu.cn; 5 Bureau of Forestry and Landscaping of Qingshen County, Meishan 620460, China; zrui547@gmail.com; 6 Guangxi Key Laboratory of Plant Conservation and Restoration Ecology in Karst Terrain, Guangxi Institute of Botany, Guangxi Zhuang Autonomous Region and Chinese Academy of Sciences, Guilin 541006, China; huang-yusong@163.com; 7 College of Forestry, Guizhou University, Guiyang 550025, China; zli7@gzu.edu.cn; 8 Jiangxi Provincial Key Laboratory of Conservation Biology, Jiangxi Agricultural University, Nanchang 330045, China

**Keywords:** China, Guangxi, plastome, phylogenetic analysis, Styracaceae

## Abstract

*Styrax chongzuoensis*, a novel endemic species with a narrow distribution in limestone regions of Chongzuo, Guangxi, China, is described herein. This new species seems somewhat similar to *Styrax fortunei*, but significantly differs from it by having long ovate or long lanceolate leaves, often with an asymmetrical base (vs. obovate-elliptic to elliptic, often with a symmetrical base) and fertile shoots with fewer flowers (1, or rarely 2 or 3, vs. many, always more than 10). Phylogenetic analyses based on the chloroplast coding sequences indicated that *S. chongzuoensis* and *Styrax japonicus* are sister taxa to each other, both clustered in the series *Cyrta* within *Styrax*. Overall, the integration of morphological and phylogenetic evidence indicates that *S. chongzuoensis* actually represents a new species. Color plates of *S. chongzuoensis* are illustrated, and a distribution map and conservation assessment of this species are also provided.

## 1. Introduction

*Styrax* Linnaeus (1753: 444) comprises ca. 140 species of trees and shrubs, mainly distributed across tropical and subtropical regions in eastern and southeastern Asia, the New World, and the Mediterranean region [1,2,3]. In China, initial records of flora recorded about 30 species and 7 varieties [4,5], which were subsequently revised to 30 species and 3 varieties in recent studies [6,7,8,9].

*Styrax* is an economically important genus, highly valued in garden landscapes for its white, fragrant flowers [10,11]. Furthermore, resin extracts from certain species have been widely used in the production of spices and medicinal products, enhancing their commercial significance [9].

As part of a comprehensive monograph study on Chinese *Styrax* species, we conducted extensive field investigations, particularly in the limestone areas of Guangxi and Yunnan provinces, where we discovered a previously undescribed species, *S. hwangiae* [3]. During subsequent fieldwork in October 2022, two shrubby *Styrax* individuals at the fruiting stage were discovered on limestone outcrops in Chongzuo, Guangxi. These plants exhibited distinctive traits, including thickly chartaceous lanceolate leaves, often with an asymmetrical base, and an infructescence bearing few fruits, which differs from all other Chinese *Styrax* species. Subsequent field observations during the flowering season were conducted to collect materials for further research. Combined morphological and molecular analyses revealed that it represents an undescribed species, which we formally propose here as *S. chongzuoensis*.

## 2. Methods and Material

### 2.1. Morphological Analysis

Living specimens of the putative new species were observed in their natural habitat within a karst mountain population in Chongzuo, Guangxi, China. The overall morphology including the plant’s habit, stems, leaves, flowers, and fruits was photographed by a digital camera and then observed and measured with a ruler. Comparisons of the new species with other *Styrax* in China and adjacent Vietnam were conducted using specimen examination and a study of the literature.

### 2.2. Species Sampling, DNA Extraction, and Data Collection

We selected 22 species of *Styrax* including our putative new species, of which 18 species from the series *Cyrta*, three species from ser. *Benzoin*, and one from ser. *Valvate*. Among all the *Styrax* species, 21 are distributed in China and Vietnam [2,3,7,8,12]. Two *Symplocos* species were designated as an outgroup. Whole-plastome sequencing was performed for *Styrax chongzuoensis*, while the complete chloroplast genome for the other species was obtained from GenBank. The voucher information and GenBank numbers of the materials used in this study are shown in Table A1.

The total genomic DNA of the new species *Styrax chongzuoensis* was extracted from silica-dried leaves using the modified CTAB method. DNA integrity was assessed via 1% (*w*/*v*) agarose gel electrophoresis, and DNA quality was evaluated using a NanoDrop spectrophotometer 2000 (Thermo Scientifc, Waltham, MA, USA). The DNA samples were subsequently sent to Novogene Bioinformatics Technology Co., Ltd. (Beijing, China), for library construction. Paired-end (2 × 150 bp) libraries were constructed using the Nova-PE150 strategy, generating over 2 Gb of genome skimming data for each sample.

The raw reads were processed using Trimmomatic v. 0.39 [13] to remove unpaired and low-depth reads, thereby enhancing the accuracy and quality of the assembly. The filtered data was assembled into the complete chloroplast (cp) genomes and nuclear ribosomal DNA (nrDNA) sequences with GetOrganelle v. 1.7.7 [14]. Bandage v.5.6.0 [15] was used to visualize the assembly results and check for circularization. After confirming circularization, the automated annotation of the complete chloroplast genome based on the reference sequence of *Styrax wuyuanensis* S. M. Hwang (MW166213) was performed using CPGAVAS2 [16], followed by manual correction with Geneious Prime v.11.0.1.

### 2.3. Phylogenetic Analysis

We imported all chloroplast sequences into Geneious Prime v.11.0.1 and extracted the coding sequences (CDS) for downstream analyses. Sequence alignment was conducted with MAFFT 7.450 [17]. Maximum likelihood (ML) analysis was implemented in IQ-TREE v2.1.3 [18], and bootstrap analysis was conducted with 20,000 ultrafast bootstraps. The best-fit BIC model was determined by ModelFinder [19]. Bayesian inference (BI) was constructed by MrBayes 3.2.6 [20] with 3,000,000 generations, sampling every 1000 generations, which was ultimately needed to ensure the assessment of convergence within the valid range (mean standard deviation of split frequency < 0.01 and effective sample size > 200). The sampled data are “burn-in” and the first 25% of the final processing are used to estimate the posterior probabilities (PPs). Percentile (MLBS) values ≥ 70 [21] indicated strong bootstrap support (MLBS), and PP values ≥ 0.95 [22] were considered strong support. Finally, the phylogenetic trees were visualized by Figtree1.4.

## 3. Results

### 3.1. General Morphology

**Figure 1:**
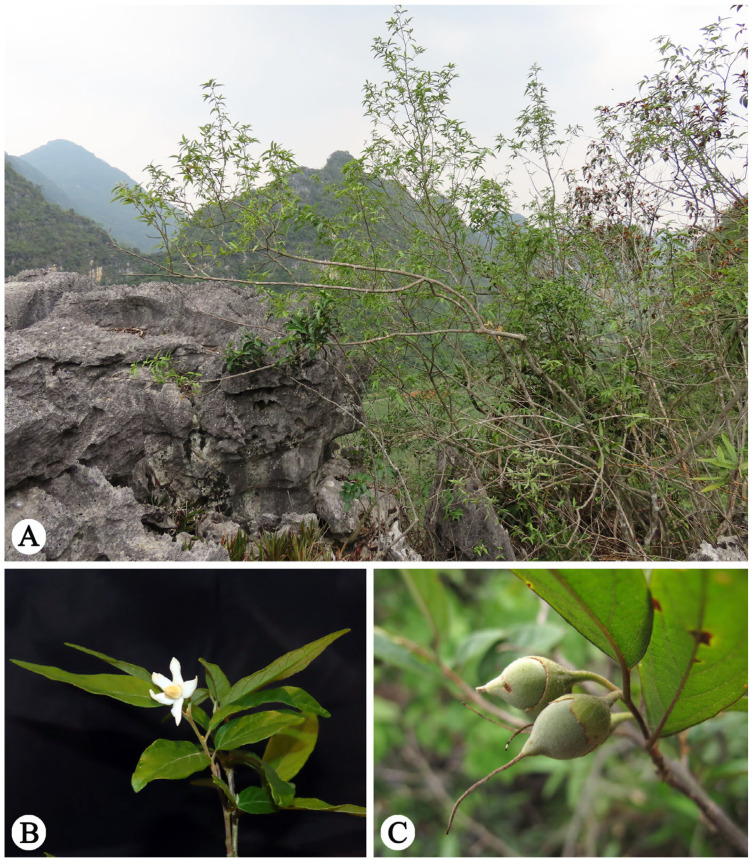
*Styrax chongzuoensis* in the wild (Jiangzhou district, Chongzuo city, Guangxi Zhuang Autonomous Region, China). (**A**) Habitat and habit; (**B**) inflorescence; (**C**) infructescence. Photographed by Weibin Xu.

**Figure 2:**
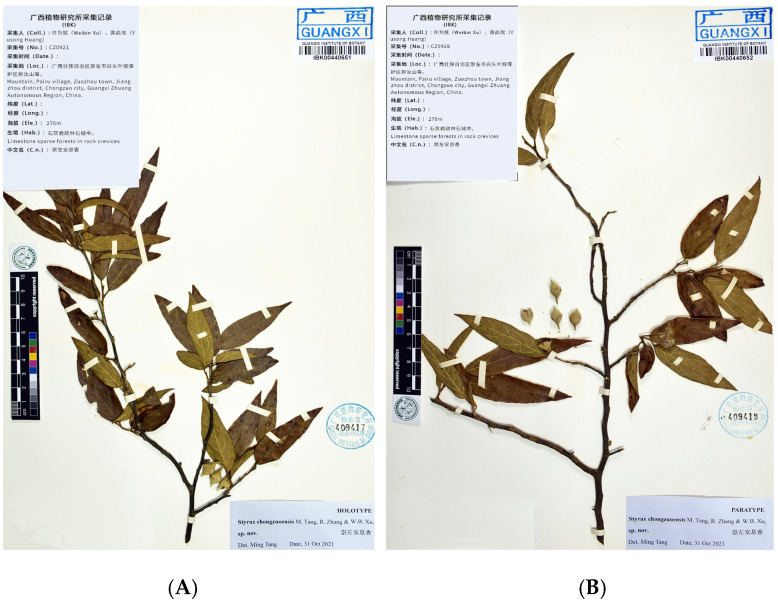
Holotype (**A**) and paratype (**B**) sheets of *Styrax chongzuoensis*. Tiandeng hill, Pairu village, Zuozhou town, Chongzuo city, Guangxi Zhuang Autonomous Region, China. *WB Xu & YS Huang CZ0921* (holotype, IBK!), *CZ0928* (paratype, IBK!).

**Figure 3:**
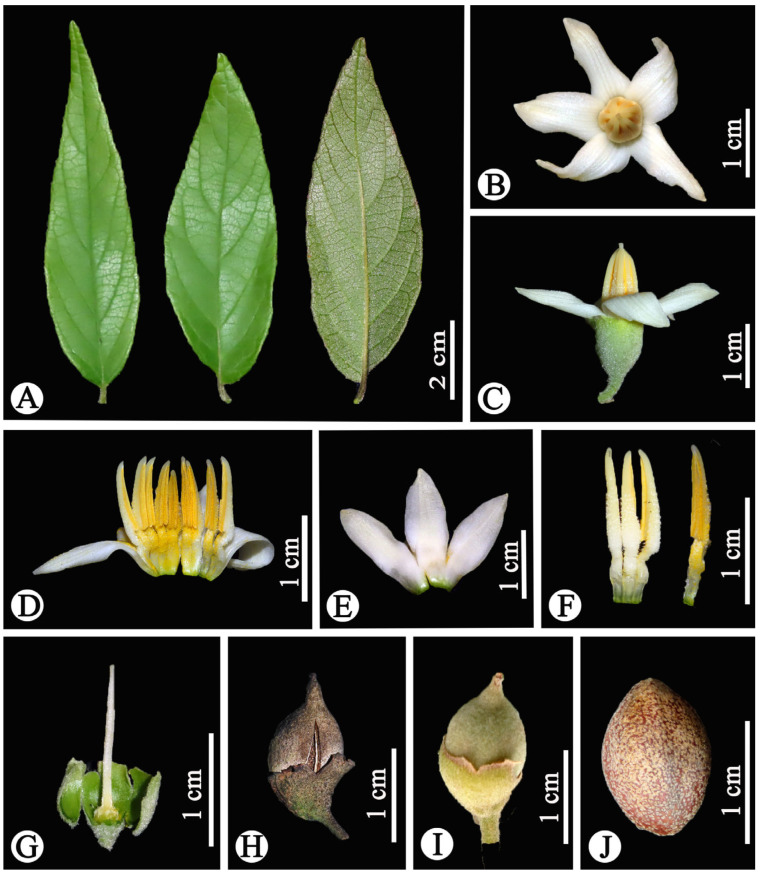
*Styrax chongzuoensis*. (**A**) Leaf blade; (**B**) flower (apical view); (**C**) flower (lateral view); (**D**) opened corolla plus androecium (ventral view); (**E**) opened corolla (abaxial view); (**F**) stamens; (**G**) pistil; (**H**) fruit (ripe); (**I**) fruit (immature); (**J**) seed. Photographed by Weibin Xu.

**Figure 4:**
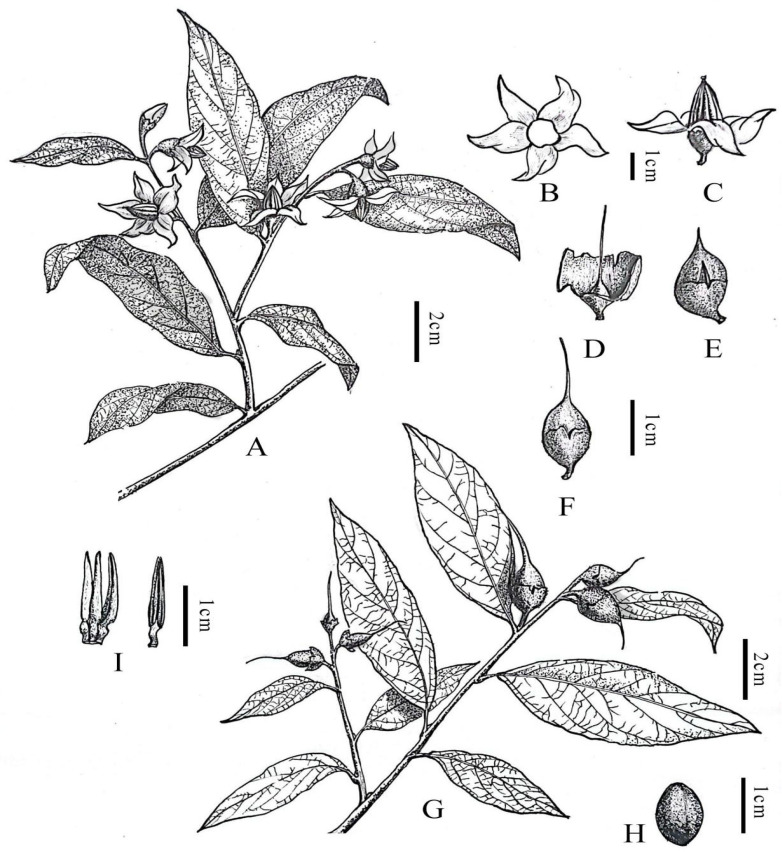
Line drawing of *Styrax chongzuoensis*. (**A**) Flowering branch; (**B**) corolla; (**C**) flower (lateral view); (**D**) sepals and stigma; (**E**) mature fruit; (**F**) fruit; (**G**) fruiting branches; (**H**) seed; (**I**) stamen. Illustration by Guoxing Peng based on living field-collected material.

**Figure 5:**
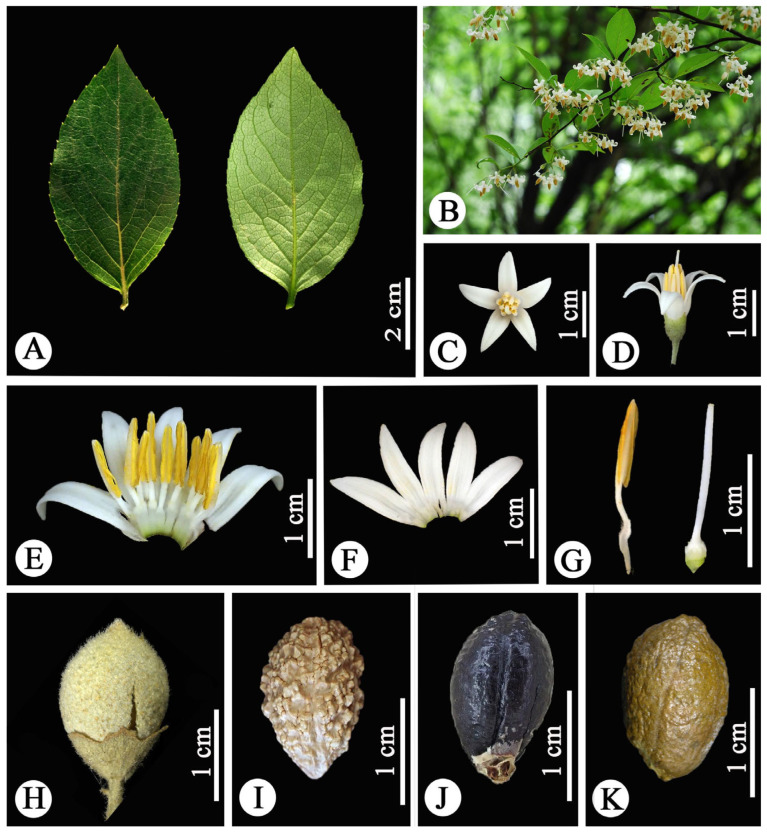
*Styrax fortune*. (**A**) Leaf blades; (**B**) inflorescence; (**C**) flower (apical view); (**D**) flower (lateral view); (**E**) opened corolla plus androecium (ventral view); (**F**) corolla lobes; (**G**) stamens (left) and pistil (right); (**H**) fruit; (**I**) seed (with tuberculous projection; lateral view); (**J**) seed (black; lateral view); (**K**) seed (brown; lateral view). Photographed by Rui Zhang.

*Styrax chongzuoensis* bears unique morphological traits within *Styrax* (Figure 1, Figure 2, Figure 3 and Figure 4*)*. It mostly resembles *S. fortunei* Hance in flower shape (Table 1). However, it shows clear differences in many morphological features. *Styrax chongzuoensis* is a shrub or small tree (≤5 m), often with many branches at the lower part, while *S. fortunei* is a larger tree (up to 20 m) with a defined trunk (Figure 5. Table 1). In addition, *S. chongzuoensis* has long ovate or long lanceolate leaf blades, often with an asymmetrical base and covered with dense stellate tomentum abaxially, while the leaf blades of *S. fortunei* are always obovate-elliptic to elliptic, with a symmetrical base, and sparsely stellate-pubescent abaxially (Figure 5. Table 1). Additionally, *Styrax chongzuoensis* often has a single flower, or sometimes two or three flowers clustering at branchlet tips, and has curved-point fruits, while *S. fortunei* often has many flowers clustering into panicles, and has round fruits with a short point (Figure 5. Table 1).

### 3.2. Phylogenetics

**Figure 6:**
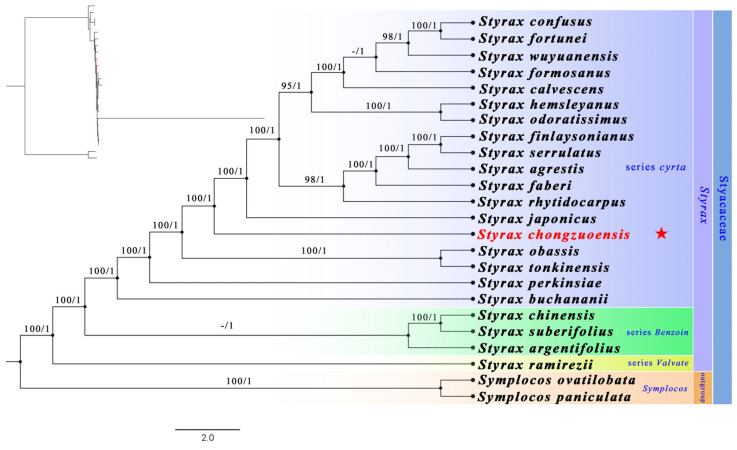
Maximum likelihood tree for the Styracaceae based on the CDS dataset, with *Styrax chongzuoensis* highlighted in red color (★). Bootstrap values (MLBS ≥ 70) and posterior probabilities (PP ≥ 0.95) are labeled above the branches.

Our maximum likelihood (ML) and Bayesian inference (BI) approaches yielded highly similar tree structures that align closely with prior research [9]. Both analyses strongly supported the placement of *Styrax chongzuoensis* within the *Styrax* sect. *Cyrta* lineage, with maximum statistical confidence (MLBS = 100%; PP = 1.00). Genetic data from chloroplast coding DNA sequences revealed that this species forms a distinct evolutionary branch that clusters closest to ser. *Benzoin* group members (Figure 6).

## 4. Taxonomic Treatment

***Styrax chongzuoensis*** M. Tang, R. Zhang & W.B. Xu, sp. nov.

Figure 1, Figure 2, Figure 3 and Figure 4

**Chinese name**: “chóng zuò ān xī xiāng” (崇左安息香)

**Type:** Tiandeng hill, Pairu village, Zuozhou town, Chongzuo city, Guangxi Zhuang Autonomous Region, China, in sparse forests on limestone karst hilltops, alt. 270 m, 27 May 2014, *WB Xu & YS Huang CZ0921* (holotype, IBK!; isotypes, IBK, JXAU!) (Figure 2).

**Diagnosis:** *Styrax chongzuoensis* is most similar to *S. fortunei* in flower shape, but could be distinguished by leaf shape and coverings (long ovate or long lanceolate, densely hairy abaxially vs. obovate-elliptic to elliptic, sparsely hairy abaxially), inflorescence (often solitary, or 2 or 3 flowers in racemes, vs. many flowers clustering in large, branched panicles), and fruit shape (with a curved point vs. fruit round with a short point).

**Description:** Deciduous shrub or small tree, 2–5 m tall; bark gray or dark gray. Leaves thickly chartaceous, narrowly long ovate or long lanceolate, 5–15 × 2.5–6.5 cm, entire, apically acuminate, base broadly cuneate, often asymmetrical, adaxially glabrous, abaxially densely yellowish-brown stellate or scalelike tomentum, lateral veins 5–8 per side; petiole 5–10 mm long, densely yellowish-brown hairs. Inflorescences terminal or axillary, often single flower, or racemes, 2 or 3 clustered at branchlet tips; peduncle, pedicel, bracteoles, and calyx densely yellowish-brown hairs; pedicel ca. 5 mm long, stellate-tomentose; bracteoles subulate or nearly subulate, ca. 3.0 mm long; calyx cup-shaped, corolla lobes 4–5, oblong, elliptical to obovate; corolla white, widely campanulate, 10–15 × 3–3.5 mm; stamens 9–11, slightly shorter than corolla, filaments curved at middle, style 10–13 mm long; fruit ovate or slightly broadly ovate, 13–17 × 8–10 mm, apiculate, cusp longer and slightly curved; seeds ovate, brown, 12–15 × 7–9 mm, rusty-brown stellate trichomes.

**Phenology:** Flowering from April to June; fruiting from August to October.

**Etymology:** This species is named after its type locality, Chongzuo city, Guangxi Zhuang Autonomous Region, China.

**Figure 7:**
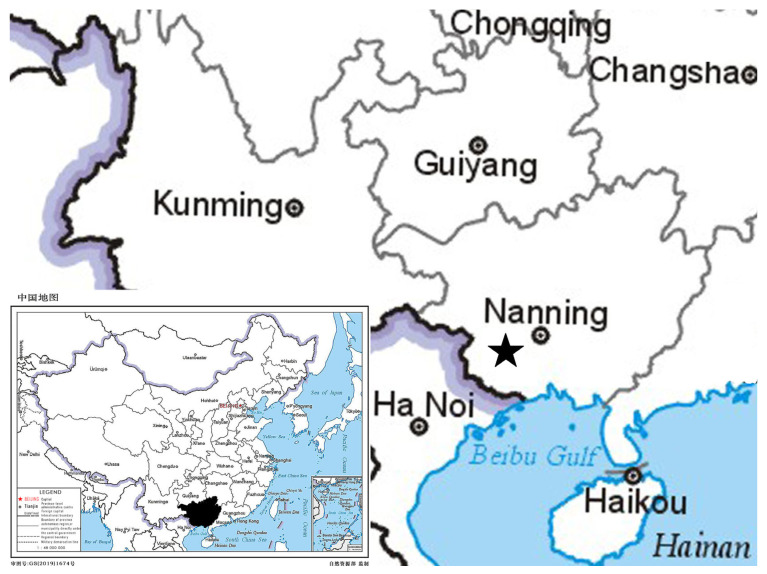
Distribution map of *Styrax chongzuoensis* (★).

**Distribution and habitat:** *Styrax chongzuoensis* is endemic to Chongzuo city, Guangxi Zhuang Autonomous Region, China (Figure 7). It grows in sparse forests of limestone in karst hill tops at altitudes of 270 m.

**Conservation status:** The Tuozhu Area, Jiangzhou District, Chongzuo city, Guangxi *White-headed Langur* Nation Reserve is the first site in China where *Styrax chongzuoensis* was recorded. It is understood that the Guangxi Chongzuo *White-headed Langur* Nation Reserve is a wildlife-type nature reserve, and the peaked rocky mountains of the reserve are mainly dominated by shrubs, small trees, and thorny vine species, with a fragmented community structure of vegetation and very fragile habitats. At present, the population of *S. chongzuoensis* was found within a radius of 1 km and does not exceed 10 mature individuals, which can only be found in the extremely narrow distribution area of the karst mountain tops in the region. However, due to the surrounding adjoining farmland and villages, the low elevation of the mountain, the very sparse vegetation, the fragile ecological environment, and the existence of a certain degree of anthropogenic interference, it may face deforestation or other unforeseen habitat disturbance and destruction; thus, the species is at a high risk of extinction, and it should be considered Critically Endangered (CR) according to the IUCN Criteria [23].

**Additional specimens examined:** Pairu Village, Zuozhou town, Chongzuo city, Guangxi Zhuang Autonomous Region, China. alt. 240 m, 13 April 2021, *WB* Xu et al. *14*,*236* (paratypes, IBK!, JXAU!); same locality. alt. 270 m, 31 October. 2021, *WB Xu & YS Huang CZ0921* (paratypes, IBK!, JXAU!).

## 5. Notes

The genus *Styrax* still likely contains undescribed or cryptic species in understudied regions, particularly karst areas that should be prioritized for botanical investigation. These limestone landscapes exhibit exceptional edaphic and topographic heterogeneity [24], creating specialized microhabitats that foster plant diversification and speciation. This is evidenced by recent discoveries of endemic taxa in Orchidaceae, *Begonia*, *Conandron*, and *Primulina* [25,26,27,28].

Recent botanical surveys in the karst regions of Guangxi and Yunnan have revealed three novel species and one new regional record within the genus *Styrax* [3]. Notably, specimens of the newly described *Styrax hwangiae* were previously misclassified as *S. chinensis* Hu & S. Y. Liang—a species widely distributed across southern China and northern Southeast Asia. However, *S. hwangiae* exhibits distinct morphological characteristics including smaller leaves, calyces with branched stellate hairs, and beaked fruit apices [3]. The Southeast Asian karst zone, particularly the areas bordering southern/western Yunnan and southwestern Guangxi, contains extensive limestone formations covering approximately 10% of the region’s landmass. This geologically unique area represents one of the world’s most significant biodiversity hotspots, harboring exceptional concentrations of endemic flora and fauna. Its distinctive ecosystem makes it particularly valuable for studying the evolutionary patterns and biogeographical distribution of *Styrax* species. Despite its ecological significance, current field research efforts in these karst habitats remain insufficient [29,30]. Our findings suggest that additional undocumented *Styrax* taxa may exist in these understudied regions, highlighting the urgent need for intensified botanical surveys focused on limestone-specific vegetation communities.

## Figures and Tables

**Table 1:** Comparison between *Styrax chongzuoensis* and *S*. *fortunei*.

Characteristic	*S. chongzuoensis*	*S. fortunei*
**Habit and Size**	Shrub or small tree (≤5 m)	Larger tree (up to 20 m)
**Leaves**	Long ovate or long lanceolate, often with asymmetrical base, densely hairy abaxially	Obovate-elliptic to elliptic, sparsely hairy abaxially
**Indumentum**	Persistent yellowish-brown trichomes on leaves/calyx	Grayish indumentum on fruits; leaves glabrescent
**Inflorescence**	Solitary, or short-racemose with 2 or 3 flowers	Paniculate, with many flowers
**Fruit**	Apiculate (curved-cusp)	Globose, shortly pointed

## Data Availability

The original contributions presented in this study are included in the article; further inquiries can be directed to the corresponding authors.

## Appendix A

**Table A1:** Voucher information and GenBank accession of species used in our study.

Accession No.	Taxon	Voucher Information	Herbarium
*	*Styrax chongzuoensis*	Xu WB et al., 14236	JXAU
MT644192	*Styrax agrestis*	Poilane E., #18626	P
PQ276582	*Styrax argentifolius*	W. T. Tsang, #30238	A
PQ276583	*Styrax buchananii*	Buchanan, E.M., #51	E
MN560141	*Styrax calvescens*	A. Henry, #721	A
MT648752	*Styrax chinensis*	-	-
MT700478	*Styrax confusus*	A. Henry, #3450	A
MT700479	*Styrax dasyanthus*	C. Silvestri, #1770	A
MN335255	*Styrax faberi*	H. F. Hance, #13738	GH
PQ276585	*Styrax finlaysonianus*	-	-
MZ285742	*Styrax formosanus*	-	-
MN733525	*Styrax hemsleyanus*	Wilson, E.H., #2574a	HBG
PP853567	*Styrax japonicus*	Nebel, O., #Herb. J. Goltz s.n. [1900-05-25]	JE
MN560143	*Styrax obassia*	-	-
MN368610	*Styrax odoratissimus*	Champion, J.G., #138	K
MZ285734	*Styrax perkinsiae*	E. H. Wilson, #2576	A
NC041138	*Styrax ramirezii*	Pringle, C.G., #6848	G/K
PQ066665	*Styrax rhytidocarpus*	-	-
MZ152917	*Styrax serrulatus*	s.coll., #s.n.	K
MZ285747	*Styrax suberifolius*	P. G. Farges, #1487	A
MZ285744	*Styrax tonkinensis*	Augustine Henry, #12006	MO
OP581012	*Styrax wuyuanensis*	-	-
MF770705	*Symplocos ovatilobata*	F. C. How, #71750	A
MG719832	*Symplocos paniculata*	Siebold, PF von, #s.n.	L

Note: “*” indicates that the data of the complete chloroplast genome has not been temporarily uploaded to NCBI.
